# Diet and Management of Artificially-Fed Children

**Published:** 1885-08

**Authors:** Addison H. Foster

**Affiliations:** College of Physicians and Surgeons of New York, 132 La Salle street; Chicago


### ON THE DIET AND MANAGEMENT OF ARTIFICIALLY FED INFANTS. *By* ADDISON H. FOSTER, M. D., (*College of Physicians and Surgeons of New York, 1866,*) *Chicago.*

(Inaugural Thesis, read before the Chicago Gynecological Seciety, 17th of July, 1885.)

This subject was not selected with the expectation of presenting anything original or exhaustive, nor with any attempt at scientific color, but to offer a few instances and results of experience as additional testimony to the value of the teachings of JACOBI, SMITH, and others, and to afford an opportunity for suggestive hints in the management of this unfortunate class of little patients.

The problem of infant food is one the general practitioner has often to meet and solve, under a sort of algebraic process of letting, first, this article of diet, and then that, represent the supposed factor of benefit, until the one filling the conditions of the case is discovered. This subject concerns, with others, especially those parents whose nervous and physical forces are more or less exhausted by the increasing social and business demands of a ceaseless metropolitan activity, and whose children are thereby too little endowed with that reserve vitalty so essential to sustain their nervous and physical balance.

Too often an apparently well-nourished child is a martyr to colic or persistent sleeplessness, from the imperfect breast-food of its devoted, but never rested mother, and it will be benefited by an artificial diet, never as digestible as nature's intended perfect food.

Infants, too, are not readily acclimated to the subtle, pernicious atmospheric influences of a populous city, and the conseqnent depression to their vitality demands an extra care in their diet, which the country-reared infant does not require. Neither has the city-fed infant an equal opportunity with its country cousin in regard to its milk. It is almost impossible to get that article pure and fresh for a majority of these bottle nurslings; some of the more favored ones occasionally do, when the parents are able to keep a healthy cow for that single purpose, but it must be stalled in a large dry barn, fed on the best of hay and grain, with pure water to drink, and if it has not plenty of yard-room for open air and exercise, must be well groomed every day.

Seven years ago, it was my lot to care for twins deprived of breast milk from their birth. Their milk was from a fine healthy cow, pastured on the prairie in the west of the city: all ways and means failed to adapt the milk to their digestive organs. The cow's condition was then changed to that noted above, and in a very few days the twins were thriving well, with the aid of "Robinson's Prepared Barley," which had, with almost every other kind of infants' food, been previously tried without success. Several weeks afterwards, while they were still in good condition, a supposed mad dog bit the cow, and the parents refused to give her milk to the infants. Fresh new milk, from single prairie-fed cows, was tried with almost diastrous bowel effects. It being mid-summer, the original cow's milk was ordered, resulting in a return to their former healthy condition within two weeks.

This individual sensitiveness to different kinds of fresh milk, especially in the season of bowel disturbances, is doubtless a common experience, but the writer cannot refrain from giving additional testimony.

Three years ago, six infants were certainly saved a long and dangerous illness, if not their lives, by finding the proper kind of milk. In one instance the relatives of a prominent druggist were offended, that their usual supply of fresh milk was criticised. "It had been brought fresh from the stone quarries every morning for two years." The patient of one year was a visitor, and suffering from a very intractable stomach and bowel attack. All remedies were useless when any of the prairie-fed milk was used. Finally they consented to a trial of milk brought from Evanston (12 miles distant), direct from the druggist's cow. In three days hardly any bowel remedies were needed, and a neighboring stall-fed cow was soon found, whose milk agreed equally well. In the same neighborhood, our highly esteemed friend and practitioner, Dr. John Bartlett, had exhausted nearly all his therapeutical resources upon a child in a similar condition, and had ordered it, as probably all of us have in a like dilemma, around the lakes. Through an accident to one of the family, this child's case came under my observation. The mother could not now make the trip, and the child's milk supply being mistrusted, the above cow's milk was advised, with the doctor's remedies continued. In less than a week there was no need of a lake trip: the former milk was fresh, but from the prairie. The same season a delicate babe with bowel trouble had baffled the healing art of a homeopathic practitioner for weeks—orthodox treatment under my hands was not more successful. The milk was from the cow, night and morning, but she was prairie-fed and watered. Finally the family was persuaded to go a mile for the nearest proper milk. The change for the better was not rapid, but it was marked and constant until complete recovery.

Permit one more case, illustrating a sensitive stomach: A delicate little girl of fifteen months, reared with a most untiring and scrupulous care in the details of management, was suddenly seized in March, 1880, with vomiting and diarrhœa, which could not be accounted for, until, by strict inquiry, it was learned that the cow, two days before, was fed from a new load of hay, which was acknowledged to be of inferior quality. The child could not continue the milk until the hay was changed for the better. Unless fresh milk is of the proper quality and agrees, we often have to resort to milk-man's milk, from the various kinds of so called "Elgin Dairy." This milk, which is always at least twenty four hours old, will agree with some healthy infants, but they are very few, in my experience, and almost never without the addition of some kind of thin gruel. With any milk, for infants under three months, Mellin's food is preferred, which is more palatable and successful than Horlick's, although supposed to be identical in composition. With Mellin's food, condensed milk occasionally agrees, but condensed milk disappoints so much more frequently than ten years ago, that it is rarely encouraged, if proper fresh milk can possibly be procured. The brand preferred is the Anglo-Swiss, or that condensed in England. In this city, the gruel probably used more than all others together, especially without a physician's advice, is that from Ridge's food. This is always encouraged, and often succeeds, from the child's birth, but should be cooked longer than the directions require.

Often the indications for Ridge's food is a tendency to looseness of the bowels, and for Mellin's food, the opposite condition. Usually that food will agree best which is most palatable to the child, though if indications demand, we can produce tolerance by the stomach, by beginning with small amounts and gradually increasing that food which has the correcting qualities.

With a few babies, till after two years of age even, the only gruel or article of nourishment, with the milk, that will possibly agree, is rice water, prepared by cooking the best rice, at least three hours. The special indication for this diet is the constant tendency to diarrhœa. In other cases it has been found as necessary to continue equally long upon "Robinson's Prepared Barley" or "Nestle's Food," "Hubbell's Prepared Wheat" or "Imperial Granum." This latter food is especially good in many cases, and with a little Mellin's food added forms the gruel now being given to three infants under my care. Oat-meal gruel is highly recommended by Dr. Jacobi, for costiveness, but it is liable, in delicate children, to make constipation more costive, or produce an aggravated form of looseness, because not easlly digested, although it often agrees with the more robust. It is necessary to use the best quality of oat-meal, which is rarely found at any of our ordinary groceries. The Akron, Ohio, brand is one of the best, and always needs to be cooked three hours at least before straining.

All understand, with Dr. Jacobi, that the gruel is partly for nourishing properties, and partly for the mechanical division of the milk particles, so that the curd shall be more porous and readily digestible.

The great aim is always so to adjust the diet, that there shall be the least possible call for drugs and medicines. In a large majority of cases, the irregularity of the bowels can be corrected by finding the proper food, and laxatives or astringents avoided.

With some infants, digestion is too tardy, and acidity with fermentation supervenes, demanding the addition of some mild stimulating aromatic tea, as that from anise seed, fennel seed or ginger, and often with some alkali, as soda or lime water, which latter has been given for fifteen months, continuously. In some cases of looseness with acidity, prepared chalk is added to each bottle of food. Half an ounce of the chalk in two ounces each of syrup and water, makes a convenient mixture from which to give a teaspoonful each time. In others with pain and frequency of passages green and curdy, the old fashioned "red tea" is given before each feeding. This tea consists of one scruple of rhubarb, two of bi-carbonate of soda, one of grated nutmeg, one-half cup boiling water and sugar to the taste; this can be bottled and one-half to one tea-spoonful given before each feeding.

For occasional gastric and intestinal disturbances, whether accompanied by dry, light-colored and offensive stools, or those frequent, thin, and mixed in character, some form of mercury is often used as an antifermentative and secretive stimulant. In the former condition, one-twentieth to one-hundreth grain of calomel, two to twenty-four hours apart, and for the latter, one-tenth to one-hundredth grain of mercury and chalk, two to eight hours apart, according to the condition of the case, will often improve the evacution in color and consistence. In the loose class it may be best to give the child chalk at the same time. Often derangements of the bowels are the results of too frequent feeding, as well as of too rich diet. There is little danger of too seldom feeding. When there is a crying demand for the bottle, the substitution of warm water, or some light aromatic decoction, two to three times daily, will often prevent the indigestion complications.

The more delicate the stomach, the more carefully must every condition of the food be studied, and when successfully adapted, must be maintained for months with the slightest possible deviation, excepting the gradual increase of the strength and quantity of the food, as the stomach will bear; for intelligent, faithful care in the minutest details of management is as essential as the kind of food; as probably all have seen, in the hands of one nurse, the same food threaten the child's destruction, that in the hands of another would bring strength, health and flesh.

In our dietetic repertory is found " Arend's Kumyss, " which even in very young infants has proved very beneficial when given alone, or when milk is added little by little until the delicate stomach can assimilate it without the kumyss.

Peptonized milk also has proved very satisfactory in the limited number of rebellious cases dealt with, and will prove a priceless boon to many an innocent sufferer from disordered bowels. The peptonizing agent used was Fairchild's *extractum* pancreatis.

The question often arises, shall the child have the mixed diet with breast-milk, when the breast supply is limited ; certainly, if the breast-milk is healthy, and agrees, for it will facilitate the digestion of the cow's milk and gruel, and keep the bowels regular and natural, but if it disagrees without the artificial food, it is not likely to agree with it.

Infants are sometimes too delicate to become nourished fully by an artificial diet, and will thrive better under daily inunctions of the best salad oil. These should be given immediately after its bath, and the bathing finished by brisk friction with the hands, moist with warm alcohol. Such infants, as well as all others in this climate, should be clothed in warm soft woolens from their birth ; and should have the invigorating influence of a well lighted room of south exposure, with as much consideration as the carefully reared house-plant.

The out-of-doors question, in any but the mildest weather, is over done and under done. The puny, bottle-fed infant, kept in a furnace heated house, bears poorly the least sudden change to a cooler air, if not fully protected, or any exposure that chills it in the least, and it speedily becomes a sufferer from congestion of the bronchial or intestinal mucous membrane. It had better remain delicately well in doors, than attempt any hardening process in the cold outside, in this capricious climate. A few moments of fresh air daily if possible is desirable, but the child must be well wrapped, and the wraps not removed too soon after coming in.

A few conclusions on the foregoing are these:

1st. The artificially fed city infant too often inherits an enfeebled nervous and physical organization.

2nd. Its surroundings are rarely of an invigorating character.

3rd. It usually has a desperate struggle for life with an imperfect diet, for even new milk from the prairie-fed Chicago cow is likely to be harmful.

4th. The wonder is, that so many escape death at the hands of over-anxious but unwise attendants.

132 La Salle street.

